# The association between interleukin-8 gene-251 A/T polymorphism and sepsis: A protocol for systematic review and meta analysis

**DOI:** 10.1097/MD.0000000000025483

**Published:** 2021-04-16

**Authors:** Shiqiao Zhao, Junzuo Gong, Songlin Yin, Xiaofeng Li, Shuangquan Zhao, Tianyi Mou, Shu Luo

**Affiliations:** Department of Emergency, Affiliated Hospital of North Sichuan Medical College, Nanchong, Sichuan Province, China.

**Keywords:** interleukin-8, meta-analysis, polymorphism, protocol, sepsis

## Abstract

**Background:**

Emerging evidence has indicated that interleukin-8 (IL-8) gene-251A/T polymorphism may affect individual susceptibility to sepsis. However, the results of published studies are inconclusive. The aim of this meta-analysis was to elucidate the association between this polymorphism and the risk and mortality of sepsis.

**Methods:**

Relevant publications were searched from PubMed, EmBase, and Web of Science databases up to January 31, 2021, with studies only in English. The reference lists of the retrieved studies were investigated as well. Pooled odds ratio (OR) with 95% confidence interval (CI) was calculated to figure out the relationship between IL-8-251 A/T polymorphisms and the risk and mortality of sepsis. All of the data were analyzed with Stata 16.0.

**Results:**

The results of this meta-analysis will be submitted to a peer-reviewed journal for publication.

**Conclusion:**

This meta-analysis will summarize the relationship between IL-8-251 A/T polymorphism and the risk and mortality of sepsis.

## 1 Introduction

Sepsis is a life-threatening organ disorder in which the host fails to control severe infection.^[1,2]^ In high-income countries, 28 million people die of septicemia each year.^[3]^ The causes of sepsis include severe infections of lung, abdomen, blood, and urethra.^[4–6]^ Among them, pulmonary infection accounts for about 64% of all cases of septicemia.^[7]^ Multiple organ dysfunction syndrome is the most common and serious complication secondary to sepsis, and it is one of the main factors of sepsis death in the world.^[8,9]^ The mortality rate of hospitalized patients with sepsis is 25% to 30%.^[10]^ Early identification could improve the prognosis of sepsis.^[11,12]^ Therefore, the treatment of patients with sepsis mainly depends on early identification and timely treatment.

The change of immune function is considered as a key factor in the pathogenesis of sepsis.^[13]^ The host immune system generates a series of substances such as cytokines in response to infection or injury. Interleukin (IL)-8 is a pro-inflammatory cytokine that is involved in the inflammatory reaction at the early stage of sepsis. Inflammation is one of the most important clinical manifestations of sepsis.^[14–16]^ During inflammation, mononuclear macrophages secrete IL-8 from the blood in tissues.^[17]^ The upregulation of IL-8 in vitro indicates the deterioration of the disease and the higher tendency to death.^[18,19]^ In addition, it has been reported that IL-8 is associated with the progression of sepsis.^[20,21]^ IL-8 blocking therapy is beneficial to the prognosis of septicemia by blocking systemic inflammatory response.

As the most common genetic variation, single nucleotide polymorphism (SNP) has attracted more and more attentions in the research on sepsis. According to previous studies, it has been obvious that single nucleotide polymorphism can predict the risk and prognosis of sepsis. It was reported that a functional SNP, -251A/T, in the promoter region of IL-8 gene can influence the expression of IL-8. Several recent studies have been carried out to explore the correlation between the SNP of IL-8–251 A/T (rs4073) polymorphism and sepsis susceptibility.^[22–27]^ However, the results were conflicting. Thus, this study was aimed to investigate the association between the IL-8-251 A/T polymorphism and the risk and mortality of sepsis by meta-analysis.

## 2 Methods

### 2.1 Study registration

The protocol of this review was registered in OSF (OSF registration number: DOI 10.17605/OSF.IO/EPR3Y), and followed the statement guidelines of preferred reporting items for systematic reviews and meta-analyses protocol^[28]^ on the basis of reports.

### 2.2 Inclusion criteria

The publications fulfilling the following criteria were included: an original study evaluating the association between IL-8-251 A/T polymorphism and the risk and/or mortality of sepsis; objects in each study were from the same epoch; including a case group of sepsis; including a control group; including precise sample size of IL-8-251 A/T polymorphism of case and control groups that could be directly extracted or calculated based on the information available.

### 2.3 Exclusion criteria

The exclusion criteria are as follows: repetition of the published studies; a meta-analysis or a review; study with insufficient or incorrect data.

### 2.4 Publication search

Two investigators independently performed a systematically computerized search for English studies through PubMed, EmBase, and Web of Science databases up to January 31, 2021. The keywords for searching were the combination of “IL-8, interleukin-8, polymorphism, sepsis, septicemia, and septic shock.” Meanwhile, references of relevant literature reviews were screened to identify potentially relevant publications. The search strategy for PubMed is illustrated in Table 1, and the corresponding keywords would be used in other databases.

**Table 1:** Search strategy in PubMed database.

Number	Search terms
#1	Sepsis[MeSH]
#2	Pyaemia[Title/Abstract]
#3	Pyemia[Title/Abstract]
#4	Pyohemia[Title/Abstract]
#5	Blood Poisoning[Title/Abstract]
#6	Poisoning, Blood[Title/Abstract]
#7	Septicemia[Title/Abstract]
#8	Severe Sepsis[Title/Abstract]
#9	Blood Poisonings[Title/Abstract]
#10	Poisonings, Blood[Title/Abstract]
#11	Pyaemias[Title/Abstract]
#12	Pyemias[Title/Abstract]
#13	Pyohemias[Title/Abstract]
#14	Sepsis, Severe[Title/Abstract]
#15	Septicemias[Title/Abstract]
#16	or/1-15
#17	Interleukin-8[MeSH]
#18	IL-8[Title/Abstract]
#19	or/17-18
#20	polymorph^∗^[Title/Abstract]
#21	susceptibility[Title/Abstract]
#22	or/20-21
#23	#16 and #19 and #22

### 2.5 Data collection and analysis

#### 2.5.1 Selection of studies

**Figure 1:**
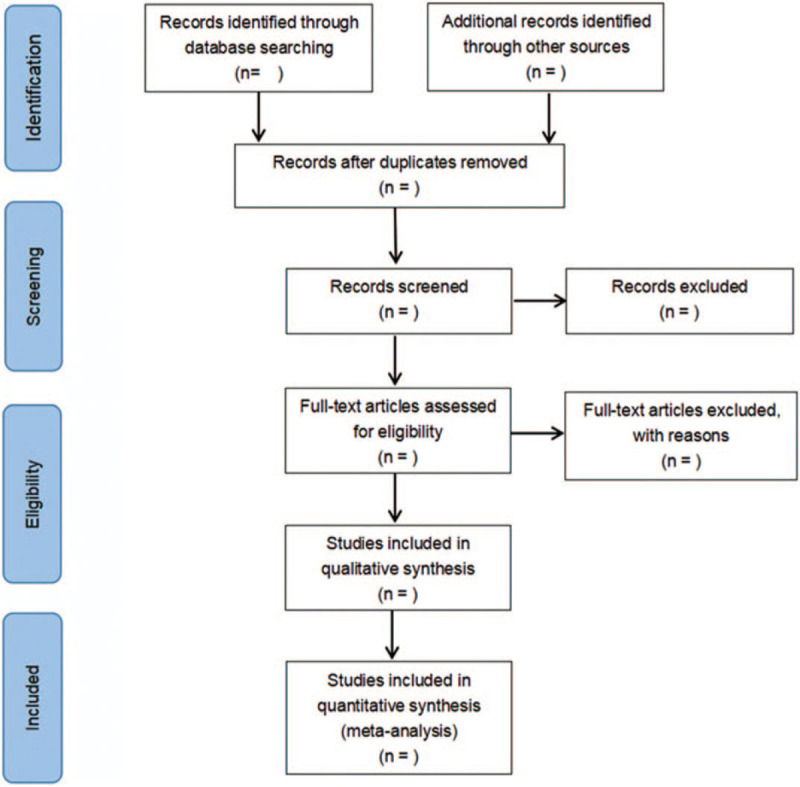
Flow diagram of study selection process.

The 2 reviewers complete the screening process independently, and any differences are decided by a third reviewer. The screening process of the article includes reading the title, the abstract, and the full text, so as to determine whether it meets the inclusion criteria. The researchers record the reasons to exclude each study in light of the preferred reporting items for systematic reviews and meta-analysis guidelines and report the screening results. The flowchart is demonstrated in Figure 1.

#### 2.5.2 Data extraction

All data were extracted by 2 independent investigators from included studies. Divergence was solved after discussion on every item. The following information was extracted from studies: first author, year of publication, country of the study and features of case and control groups such as ethnicity, age group, type of case and controls, genotype frequencies, genotyping method, and *P* value for Hardy–Weinberg equilibrium (HWE) of controls. In some studies, the risk and mortality of sepsis are discussed. In this case, the information was collected.

#### 2.5.3 Methodology quality assessment

According to Newcastle-Ottawa Scale (NOS) by 2 researchers, the quality of included studies was evaluated.^[29]^ Available data were extracted by 2 authors independently. Disagreements were dealt with by a third reviewer. The NOS values arrange from 0 to 9. Studies with the score of 6 are considered to be of high quality.^[30]^

#### 2.5.4 Dealing with missing data

The reason for the loss of data in the period of data screening and extraction is identified here. We would attempt to contact the authors if the data of potential studies are insufficient, missing, or vague. These studies would be excluded only if the data are not available through the method described above.

#### 2.5.5 Statistical analysis

The HWE for control subjects of each studies was evaluated by conducting a chi-square test, and *P* < .05 was seen as significant disequilibrium. Odds ratios (ORs) and the 95% confidence intervals (95% CIs) were calculated to evaluate the association between IL-8-251 A/T polymorphism and the risk or mortality of sepsis. The pooled ORs were executed for homozygote comparison, dominant and recessive models, allele comparison and heterozygote comparison. The heterogeneity was calculated by performing the *χ*^2^-based *I*^2^ test and the Q test. The fixed-effect model (the Mantel-Haenszel method) was chosen when the *I*^2^ value is <50%. Although the *I*^2^ is >50%, a random-effects model (DerSimonian and Laird method) was adopted. All of the statistical analyses were conducted by the STATA 16.0 (StataCorp, College Station, TX), and the *P* values were 2-sided. 2.5.6. Subgroup analysis

In the analysis on sepsis risk, subgroup analyses based on age group, ethnicity, restricted healthy controls were performed. Subgroup analysis on age and ethnicity in the mortality analysis was also made.

#### 2.5.6 Sensitivity analysis

The eligible study was sequentially removed to perform the sensitivity analysis.

#### 2.5.7 Assessment of publication biases

Publication bias was assessed by Begg rank correlation and Egger linear regression. The publication bias was regarded as statistically significance when *P* < .05.^[31,32]^

#### 2.5.8 Ethics and dissemination

The content of this article does not involve moral approval or ethical review and would be presented in print or at relevant conferences.

## 3 Discussion

IL-8 belongs to the COX2C subfamily of inflammatory mediators and participates in the regulation of inflammatory acute phase.^[33]^ Studies have revealed that the promoter of IL-8 gene-251 A/T allele can upregulate the expression of IL-8 through transcriptional regulation.^[34]^ Therefore, it plays an important role in the pathogenesis and progression of sepsis

The association between IL-8-251 A/T polymorphism and the risk or mortality of sepsis has been widely investigated.^[22–27]^ Because of the controversial findings, a meta-analysis is necessary to bring some new insights into this topic. This study has the largest sample so far to address this issue.

This study has the following shortcomings. First, due to a change in the definition of sepsis, the selected study is unlikely to have the same definition, as our analysis includes studies over a longer period of time. Second, most of the subjects of the study are whites, and more researches are needed to further study other races. Sepsis is a complex disease, and its pathogenic factors include genetic and environmental factors. The results of this study did not adjust other confounding factors, so the interpretation of the results should be cautious. In spite of this, this study still ensures the truthfulness and reliability of the research results from the following points. On the one hand, this paper includes a large number of cases and controls from different studies, which significantly increases the testing efficiency of statistics. On the other hand, for the same population source or repeatedly published literature, only the larger or recent data results are included in the study to ensure that there is no obvious selection bias.

In summary, this meta-analysis will summarize the relationship between the IL-8-251 A/T polymorphism and the risk and mortality of sepsis. However, considering the limitations of this study, better design and larger sample size are needed to confirm this conclusion.

## Author contributions

Data curation: Shiqiao Zhao and Junzuo Gong.

Formal analysis: Junzuo Gong.

Methodology: Junzuo Gong.

Project administration: Shu Luo.

Supervision: Songlin Yin.

Validation: Xiaofeng Li and Tianyi Mou.

Visualization and software: Shuangquan Zhao.

Writing – original draft: Shiqiao Zhao, Junzuo Gong and Shu Luo.

Writing – review & editing: Shiqiao Zhao, Junzuo Gong and Shu Luo.

## Author contributions

**Conceptualization:** Shu Luo, Shiqiao Zhao.

**Funding acquisition:** Shu Luo.

**Project administration:** Shu Luo, Shiqiao Zhao.

**Writing – original draft:** Shu Luo, Shiqiao Zhao, Junzuo Gong.

**Writing – review & editing:** Shu Luo, Shiqiao Zhao, Junzuo Gong.

**Data curation:** Shiqiao Zhao.

**Investigation:** Junzuo Gong.

**Resources:** Songlin Yin.

**Software:** Songlin Yin.

**Supervision:** Songlin Yin.

**Validation:** Xiaofeng Li.

**Visualization:** Shuangquan Zhao, Tianyi Mou.
